# Differential expression of beta-catenin and dickkopf-1 in the third trimester placentas from normal and preeclamptic pregnancies: a comparative study

**DOI:** 10.1186/1477-7827-11-17

**Published:** 2013-03-04

**Authors:** Zhan Zhang, He Li, Linlin Zhang, Liting Jia, Peng Wang

**Affiliations:** 1 Department of Clinical Laboratory, The Third Affiliated Hospital of Zhengzhou University, Zhengzhou, Henan, China; 2 Department of Obstetrics and Gynecology, The Third Affiliated Hospital of Zhengzhou University, Zhengzhou, Henan, China

**Keywords:** Severe preeclampsia, Beta-catenin, Dickkof-1, Placenta

## Abstract

**Background:**

Beta-catenin is a key nuclear effector of Wnt signaling which could be antagonized by dickkopf-1(DKK1). Beta-catenin and DKK1 are involved in a variety of biological processes; however, their expression in the placenta with severe preeclampsia (PE) has not been elucidated. This study was aimed to detect the localization and compare the expression of beta-catenin and DKK1 in normal and preeclamptic placenta.

**Methods:**

Sixty pregnant women who underwent cesarean section were enrolled in this study, including 30 healthy pregnant women in the control group and 30 preeclamptic women in the severe PE group. Real-time polymerase chain reaction (real-time-PCR) and western blot were employed to detect the beta-catenin and DKK1 mRNA and protein expression levels, respectively, and their locations were evaluated by immunohistochemistry (IHC).

**Results:**

Our results indicated that beta-catenin and DKK1 were expressed predominantly in the syncytiotrophoblast and the extravillous trophoblast (EVT). The beta-catenin mRNA and protein expressions were significantly decreased, whereas the DKK1 significantly increased in preeclamptic placental tissues compared to normal placental controls.

**Conclusions:**

In conclusion, decreased beta-catenin expression, as well as DKK1 over-expression might be associated with the process of the pathogenesis of PE. Further studies would elucidate their exact roles in the pathogenesis of PE.

## Background

Preeclampsia (PE) is a common, pregnancy-specific disease that belongs to the family of hypertensive disorders in pregnancy and is characterized by new onset of hypertension and proteinuria after 20 weeks of gestation. PE is a major contributor to maternal and fetal morbidity and mortality [1]. The precise mechanisms of PE pathogenesis remain unknown. Many literatures indicated that the placenta is the central organ in the pathogenesis of PE, and it is a widely accepted hypothesis that placenta dysfunction may contribute to the process of PE. Trophoblasts are the primary cell types found in the placenta. The normal differentiation, proliferation, migration and invasion ability of trophoblasts are crucial to the placentation. However, some of the placental abnormalities, including deficient implantation, abnormal trophoblast invasion of spiral arterioles, and improper placental vascular development, are believed to lead to PE [2,3]. There are multiple signaling pathways involved in mediation trophoblasts function during the placentation process, the pathological of this process is very complicated, the precise mechanism has not yet fully understood. Many recent research are studying about the Wnt pathway in human pregnancy as well as pregnancy complications.

Wnt signaling has been identified as an essential pathway which can direct cell proliferation, migration, and tissue homeostasis. The canonical Wnt signaling is activated when a Wnt ligand binds to the Frizzled receptors and their co-receptors. The signal is then transmitted into the cytoplasm by a series of cellular factors, and these events lead to the stabilization of β-catenin in the cytoplasm. Consequently, β-catenin accumulates and travels into the nucleus to form complexes with T cell-specific factors (TCFs)/lymphoid enhancer-binding factor-1(LEF-1), and activate target gene expression [4-6]. β-catenin is the primary Wnt effector, which serves as a coactivator through its ability to recruit the components that promote chromatin remodeling and transcriptional initiation/elongation [7]. Dickkopf-1 (DKK1) is a secreted glycoprotein that can antagonize the canonical Wnt signaling pathway, and this cascade influences numerous biological processes [8,9]. Accumulating evidences suggest that Wnt signaling has been identified as a pivotal pathway that promotes endometrial function, decidualization, trophoblast differentiation and invasion, and an inappropriate activation of the Wnt signaling is often associated with severe manifestations of human disease [10-13]. We speculated that there might be an association between abnormal expression of Wnt signaling and PE. Hence, in this study, we employed the real-time PCR, immunohistochemistry (IHC) and western blot to detect the expression of β-catenin and DKK1 in the placenta from normal and preeclamptic pregnancies.

## Methods

### Subjects and sample collection

Sixty pregnant women who had undergone cesarean section at the Third Affiliated Hospital of Zhengzhou University, from January 2010 to January 2012, were enrolled in this study. This sample included 30 normal pregnant women, who constituted the control group, and 30 preeclamptic women, who were classified as the severe PE group. This study was approved by the Ethics Committee of Zhengzhou University School of the Third Clinical Medicine, China. The informed consent was obtained from all subjects. The criteria for diagnosis of severe PE were strictly based on the American College of Obstetricians and Gynecologists Practice Bulletin (ACOG 2002) [14]. Normal pregnancy was defined as pregnancy characterized by normal blood pressure values (<140/90 mmHg) and negative proteinuria. Subjects with diabetes mellitus, chronic hypertension, renal disease, polycystic ovarian syndrome, multiple gestations and fetal malformations were excluded from this study. The patient demographic characteristics are summarized in Table 1.

**Table 1:** Demographic characteristics for normal and preeclamptic pregnancies

**Variables**	**N (n=30)**	**sPE (n=30)**	**P Value**
*Maternal age (Years)*	30.73±3.35	29.17±5.48	0.188
*Gestational age (weeks)*	37.15±0.96	36.63±1.08	0.056
*Maternal BMI (kg/m2)*	29.06±3.68	29.50±3.87	0.653
*Systolic pressure (mmHg)*	112.37±9.02	154.20±12.92	0.000 ^a^
*Diastolic pressure (mmHg)*	74.97±7.37	101.23±10.20	0.000 ^a^
*Neonatal weight (g)*	3248.37±529.03	2240.0±729.71	0.000 ^a^

Normal control group *(N)*, severe preeclampsia group *(sPE)*. Data presented as mean ± SD.

^a^ Significant at P < 0.05.

The placental biopsies were collected from the maternal aspect of the placenta within 15 min of delivery. Five small separate biopsies (2 cm × 2 cm × 1 cm) were excised from the placental center, as well as from each quadrant (3, 6, 9, and 12 o’clock of the placenta) to avoid sampling bias. Placenta sampling included full-thickness placental blocks biopsies were not collected from the placental periphery or areas of calcification and necrosis. A portion of the samples were fixed in 10% formalin for immunohistochemistry, and the other portion of samples were flash frozen in liquid nitrogen and stored at −80°C until the tissue was processed. In this study, these samples were studied pooled.

### RNA extraction and real-time RT-PCR

The total RNA was extracted from placental biopsies using the Trizol reagent (Invitrogen, Carlsbad, CA) according to the manufacturer's protocol. RNA was fractionated on agarose/ethidium bromide gels to confirm the integrity. cDNA synthesis was performed using a Reverse Transcriptase M-MLV kit as per the manufacturer’s instructions (Takara, Dalian, China). Quantitative real-time PCR assays of β-catenin and DKK1 were performed using the Ultra SYBR Mixture (with ROX) (CWBIO, Beijing, China) in an ABI 7500 Fast Real-time PCR System (ABI, Foster City, CA). The PCR primers were designed and synthesized by Sangon Biotech (Shanghai, China); utilized primers are shown in Table 2.

**Table 2:** Primers used for realtime-PCR quantifications

**Gene name**	**Product size**	**Direction**	**Primer sequence**
*β-catenin*	175bp	Forward	TTCGCCTTCACTATGGACTACC
		Reverse	GCACGAACAAGCAACTGAACTA
*DKK1*	196bp	Forward	TAGCACCTTGGATGGGTATTC
		Reverse	GCCTTTTCTCCTATGCTTGGT
*GAPDH*	116bp	Forward	TCGTGGAAGGACTCATGACC
		Reverse	AGGGATGATGTTCTGGAGAG

The real-time PCR was performed in a final volume of 20 μl, which contained 10 μl of 2×Ultra SYBR Mixture (With ROX), 0.4 μl of forward and reverse primers, respectively, 1 μl of template cDNA, and RNA free H_2_O 8.2 μl to compose the final volume. A sample without cDNA was subjected to an identical protocol as a negative control. The PCR amplification was accomplished with initial denaturation at 95°C for 10 min, followed by 35 cycles at 95°C for 20 s and 60°C for 1 min. During the melt cycle, the temperature was increased by increments of 1°C from 60°C to 95°C. The CT values for the targets (β-catenin and DKK1) and GAPDH genes were provided by real-time PCR instrumentation. The comparative method 2^-ΔΔCT^ was used for the relative quantification of β-catenin and DKK1 transcription between the control and the severe PE groups.

### Immunohistochemistry

The fixed biopsies were deparaffinized, and the paraffin blocks were cut into 4 μm sections and mounted onto microscope slides. The sections were then hydrated by sequential immersion in xylene and graded alcohol solutions. Prior to staining, antigen retrieval was accomplished by boiling tissue slides in a citrate buffer solution. Endogenous peroxidase was quenched with 3% hydrogen peroxide for 20 min. After blocking the tissue with goat serum, the sections were incubated for 1 h at room temperature with the primary antibodies specific to β-catenin (1:600 dilution, ab6302, Abcam), DKK1(1:300 dilution, ab88334, Abcam) and HLA-G (1:100 dilution, MA1-19219, Thermo scientific, Rockford, CA). The phenotype characteristic of EVT was confirmed with the use of serial sections stained with HLA-G. Negative control sections were incubated for 1 h at room temperature with phosphate buffer solution (PBS). The polink-2 plus® polymer HRP detection system for rabbit and mouse primary antibody kits and DAB detection kit (Zsbio, Beijing, China) were used following the manufacturer' s protocols. Stained slides were examined with an Olympus microscope (Olympus IX71, Tokyo, Japan). Images for analysis were captured by a digital camera using Image Pro 6.0 software.

The sections were assessed by two observers separately. The immunohistochemical staining was graded on a semiquantitative scale. Briefly, staining intensities were documented according to the following categories: 0(absent staining/ no color), 1(weak staining/pale brown color), 2 (distinct staining/dark brown color), 3 (strong staining/brownish-black color).

### Western blot analyses

The frozen placental tissue was directly homogenized with the use of RIPA buffer containing a protease inhibitor cocktail (Solarbio, Beijing, China). The total protein concentration was determined with a BCA assay (Sangon, Shanghai, China). Following quantification, the samples containing 100 μg of protein were separated by 10% SDS polyacrylamide gel electrophoresis, and then they were electrophoretically transferred to the nitrocellulose membranes. The membranes were blocked for 90 min at room temperature with blocking buffer (5% skim milk, 0.1% Tween 20, 0.01M TBS) and incubated overnight at 4°C with mouse monoclonal to DKK1 (1:600 dilution, ab88334, Abcam) and rabbit polyclonal to β-catenin (1:4000 dilution, ab6302, Abcam), respectively. Then the membranes were incubated for 1 h at room temperature with their respective secondary antibodies. The peroxidase-conjugated goat anti-mouse IgG and the goat anti-rabbit IgG were purchased from Dingguo Biotech (1:15000 dilution, Beijing, China). The chemiluminescent detection was performed using a Pro-light HRP chemiluminescent detection kit (Tiangen, Beijing, China). Image J analysis software was utilized to estimate the relative density of the proteins of interest. β-actin was detected by rabbit polyclonal anti-β-actin antibody (1:5000 dilution, Bioss, Beijing, China), and the expression of β-actin was used for verifying the protein loading variations.

### Statistical analysis

All statistical analyses were performed using SPSS 17.0 software. Quantitative data are presented as mean ± standard deviation (SD). Comparison of two groups was performed using either unpaired t test (for parametric data) or the Mann–Whitney U-test (for non-parametric data). The differences in enumeration data were detected with the χ2 test. The 2^-ΔΔCT^ method [15] was used to analyze the relative gene expression from real time PCR data. Differences were considered to be statistically significant when P< 0.05.

## Results

### Reduced β-catenin mRNA expression and increased DKK1 mRNA expression in severe PE

**Figure 1:**
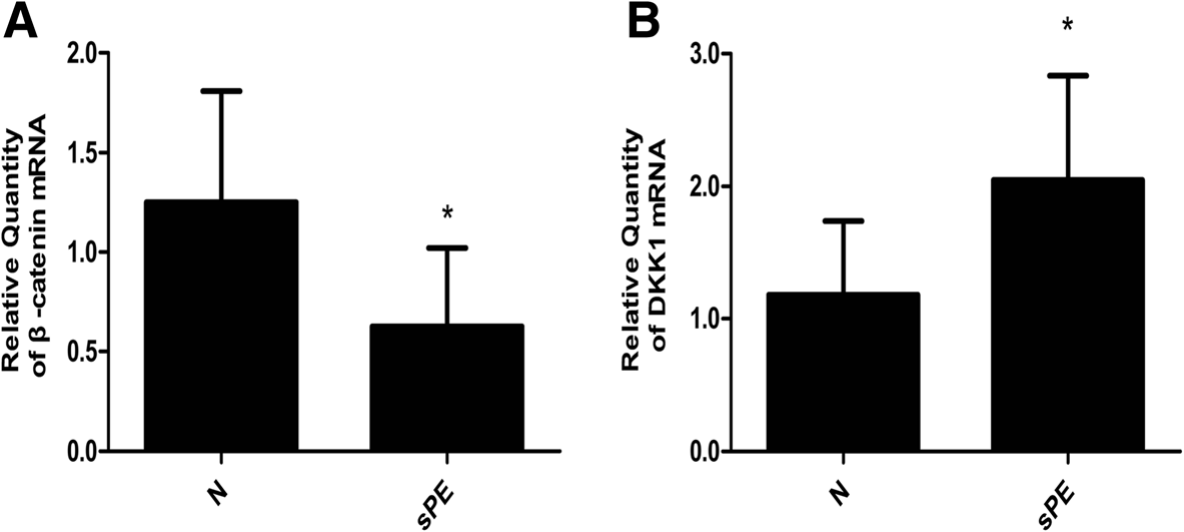
**Relative quantity of β-catenin and DKK1 mRNA expression in normal and severe preeclampsia group.** Representative data are shown: (**A**) the relative quantity of β-catenin mRNA expression. (**B**) The relative quantity of DKK1 mRNA expression. Results indicated that β-catenin and DKK1 mRNA expression could be detected in both the normal control (N) and severe preeclampsia (sPE) groups. Compared with the control group, the β-catenin mRNA expression was significantly decreased and the DKK1 mRNA expression was significantly increased in sPE group (p<0.05). (Data are presented mean ± SD, n=30 for each group, *p < 0.05, respectively).

We employed real-time PCR to examine relative quantity of β-catenin and DKK1 mRNA in both groups (n=30 for each group). Our results indicated that β-catenin and DKK1 mRNA expression could be detected in both the severe PE and normal control groups, The β-catenin mRNA expression was decreased in the severe PE group(0.63±0.39) compared with the control group (1.25±0.56) (P<0.05) (Figure 1A). In contrast, the DKK1 mRNA expression of severe PE group (2.05±0.78) was significantly increased compared with the control group (1.18±0.56) (P<0.05) (Figure 1B).

### Localization of β-catenin and DKK1 protein expression in the placenta during the third trimester

**Figure 2:**
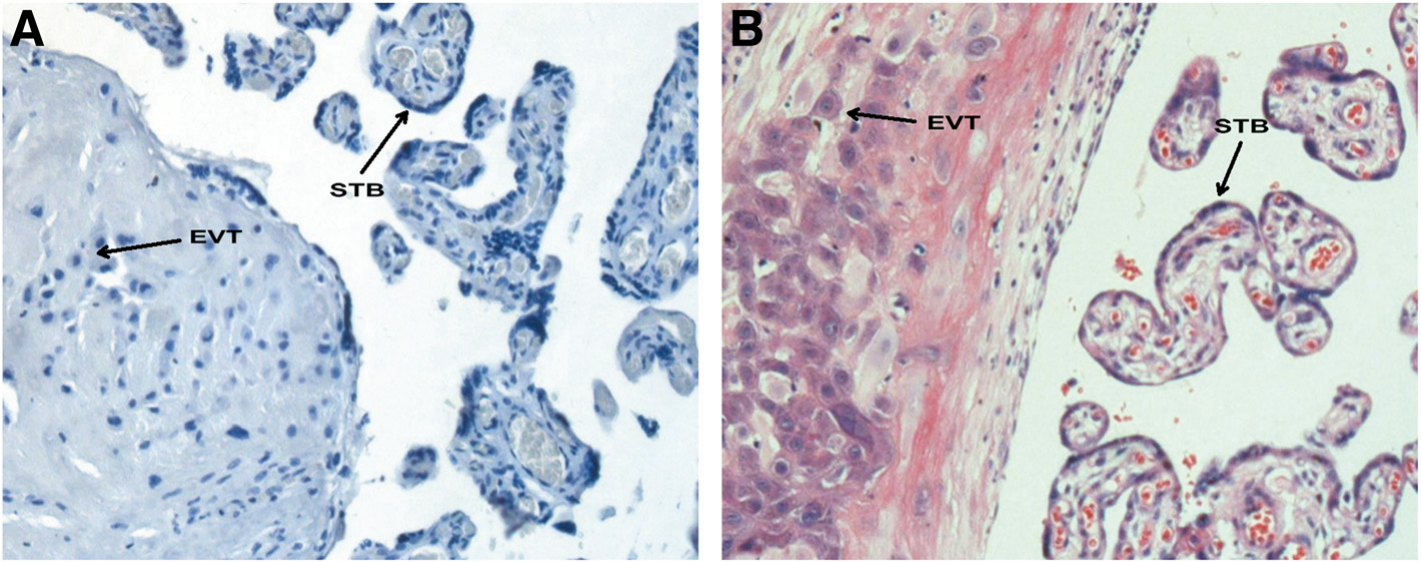
**The PBS staining and the H&E staining of placental tissue sections.** Figure 2A shows the PBS staining as negative control, Figure 2B shows the H&E staining. STB = syncytiotrophoblast, EVT = extravillous trophoblast. Original magnification: 200× for **A** and **B**.

To assess the presence of β-catenin and DKK1 protein in the placental tissue during the third trimester, immunohistochemical analyses were performed. β-catenin and DKK1 immunostaining were examined in sections from 40 placentas (n=20 for each group). The sections were examined by hematoxylin and eosin (H&E) staining before IHC analysis. The image of negative control section was shown in Figure 2A. H&E staining was shown in Figure 2B.

**Figure 3:**
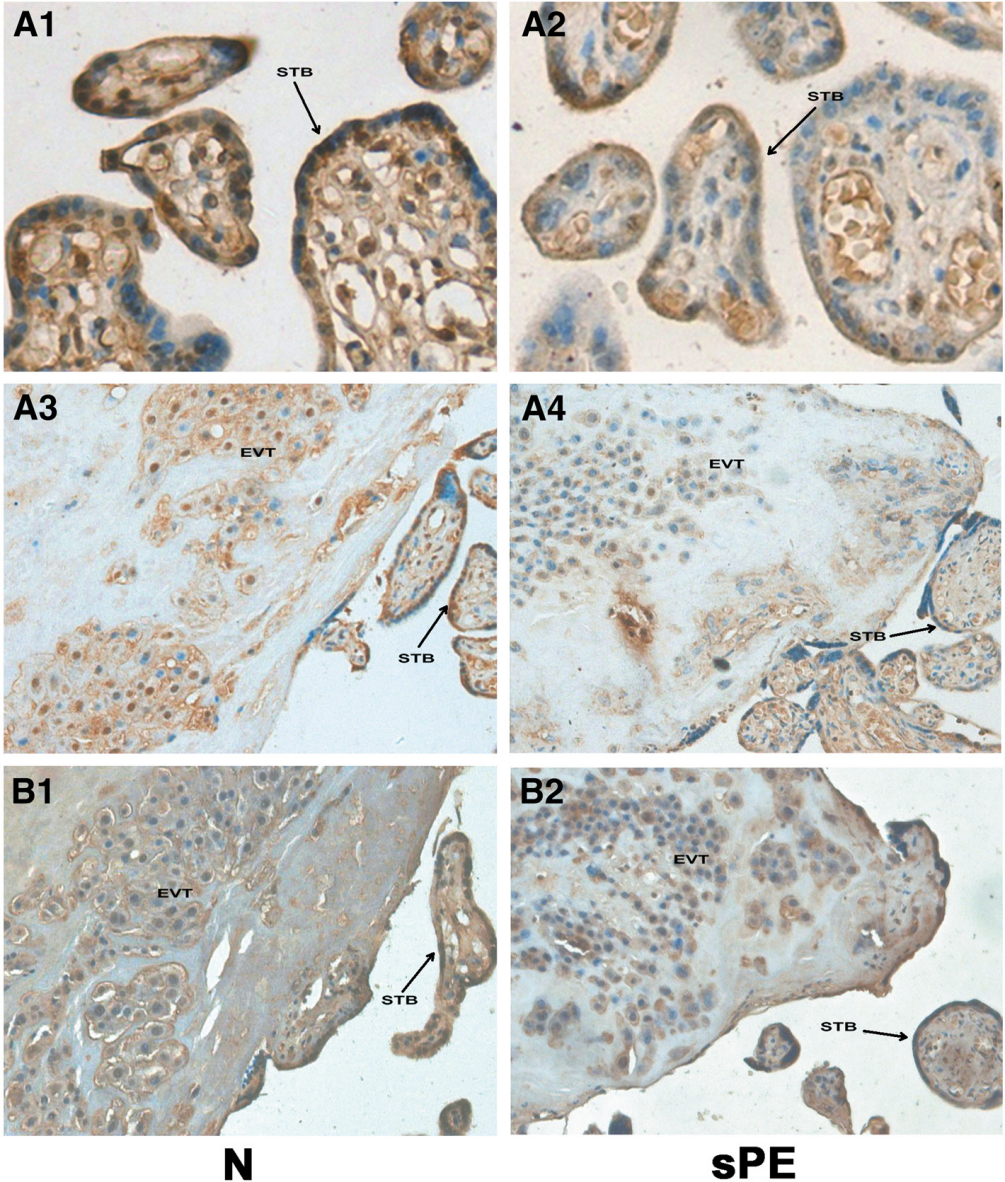
**Immunostaining of β-catenin in the placental tissue sections from normal and severe preeclampsia groups.** Figure 3 A1 and A3 show the immunostaining of the β-catenin in normal control (N) group; A2 and A4 show the immunostaining of the β-catenin in severe preeclampsia (sPE) group. The staining intensity of β-catenin in the placental tissue of the severe PE group (A2 and A4) was weaker than the control group (A1 and A3). B1 and B2: serial sections of A3 and A4 stained with HLA-G. STB = syncytiotrophoblast, EVT = extravillous trophoblast. Original magnification: 400× for A1 and A2, 200× for A3, A4, B1, B2.

**Figure 4:**
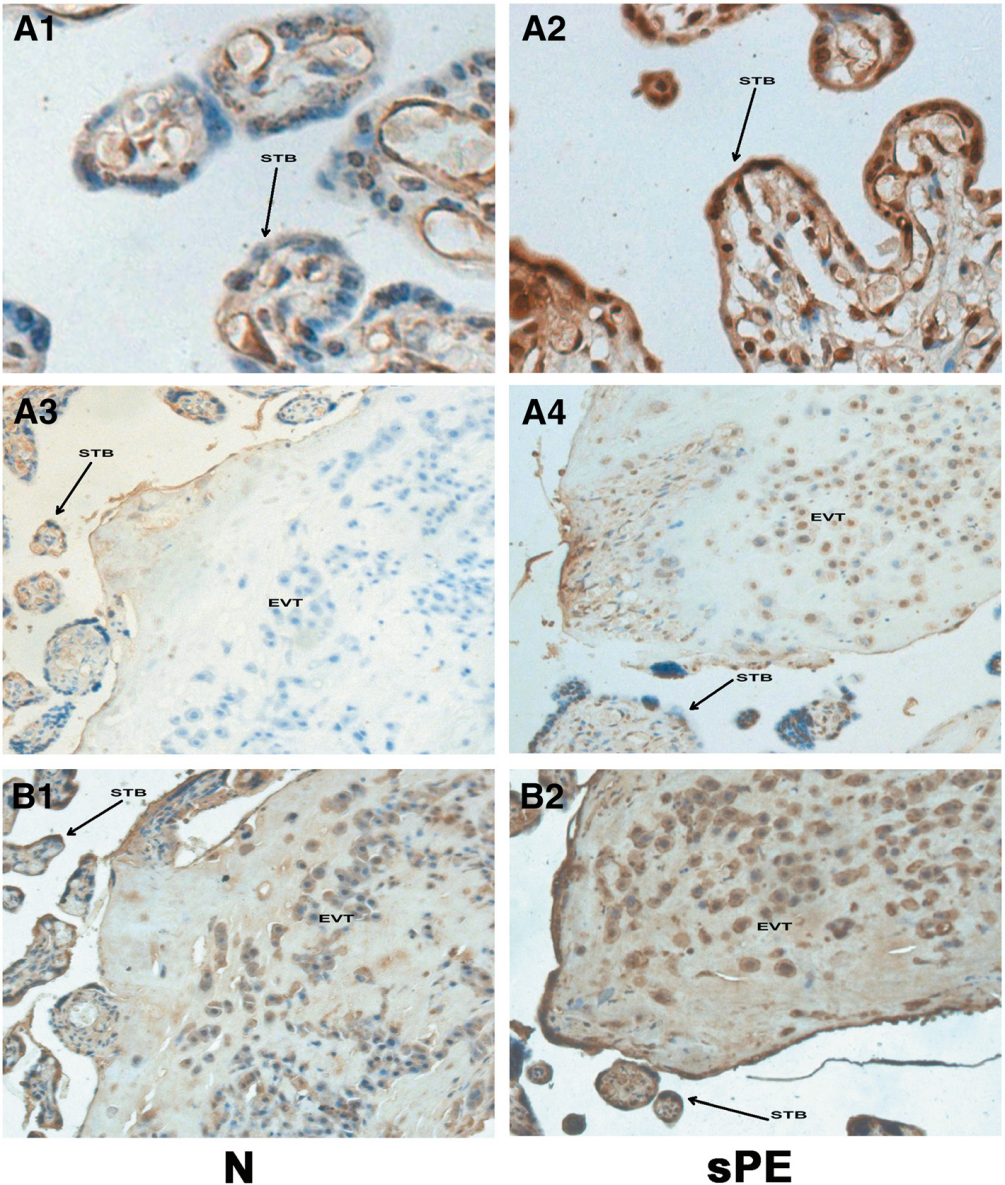
**Immunostaining of DKK1 in the placental tissue sections from normal and severe preeclampsia groups.** Figure 4 A1 and A3 show the immunostaining of the DKK1 in normal control (N) group; A2 and A4 show the immunostaining of the DKK1 in severe preeclampsia (sPE) group. The staining intensity of DKK1 in the placental tissue of the severe PE group (A2 and A4) was greater than the control group (A1 and A3). B1 and B2: serial sections of A3 and A4 stained with HLA-G. STB = syncytiotrophoblast, EVT = extravillous trophoblast. Original magnification: 400× for A1 and A2, 200× for A3, A4, B1, B2.

The immunohistochemical staining for the β-catenin and DKK1 proteins was observed in the syncytiotrophoblast and extravillous trophoblasts (EVT). The phenotype characteristic of EVT was confirmed with the use of serial sections stained with HLA-G. Our results indicated that the staining intensity of β-catenin in the placental tissue of the severe PE group (Figure 3 A2 and A4) was weaker than the control group (Figure 3 A1 and A3). Figure 3 B1 and B2 were serial sections of A3 and A4, which stained with HLA-G. However, the DKK1 showed the opposite pattern, with greater staining in the severe PE group (Figure 4 A2 and A4) compared to the control group (Figure 4 A1 and A3). Figure 4 B1 and B2 were serial sections of A3 and A4, which stained with HLA-G.

Table 3 and Table 4 summarized the results which were categorized by the intensity of immunostaining. The staining intensity of β-catenin in the placental tissue of the severe PE group was weaker than the normal controls (χ2 =17.107; P < 0.001), while the staining intensity of DKK1 was greater in the severe PE group (χ2 =10.093; P = 0.001).

**Table 3:** The immunostaining of β-catenin in severe PE group and normal control group

**Immunostaining of β-catenin**	**Absent staining**	**Weak staining**	**Distinct staining**	**Strong staining**	***χ2***	**P**
*N (n=20)*	0	2	16	2	17.107	0.000 ^a^
*sPE (n=20)*	2	13	5	0		

Normal control group *(N)*, severe preeclampsia group *(sPE)*.

^a^ Significant at P < 0.05.

**Table 4:** The immunostaining of DKK1 in severe PE group and normal control group

**Immunostaining of DKK1**	**Absent staining**	**Weak staining**	**Distinct staining**	**Strong staining**	***χ2***	**P**
*N (n=20)*	2	10	8	0	10.093	0.001 ^a^
*sPE (n=20)*	0	3	14	3		

Normal control group *(N)*, severe preeclampsia group *(sPE)*.

^a^ Significant at P < 0.05.

### The differential expression of β-catenin and DKK1 protein in control and severe PE group

**Figure 5:**
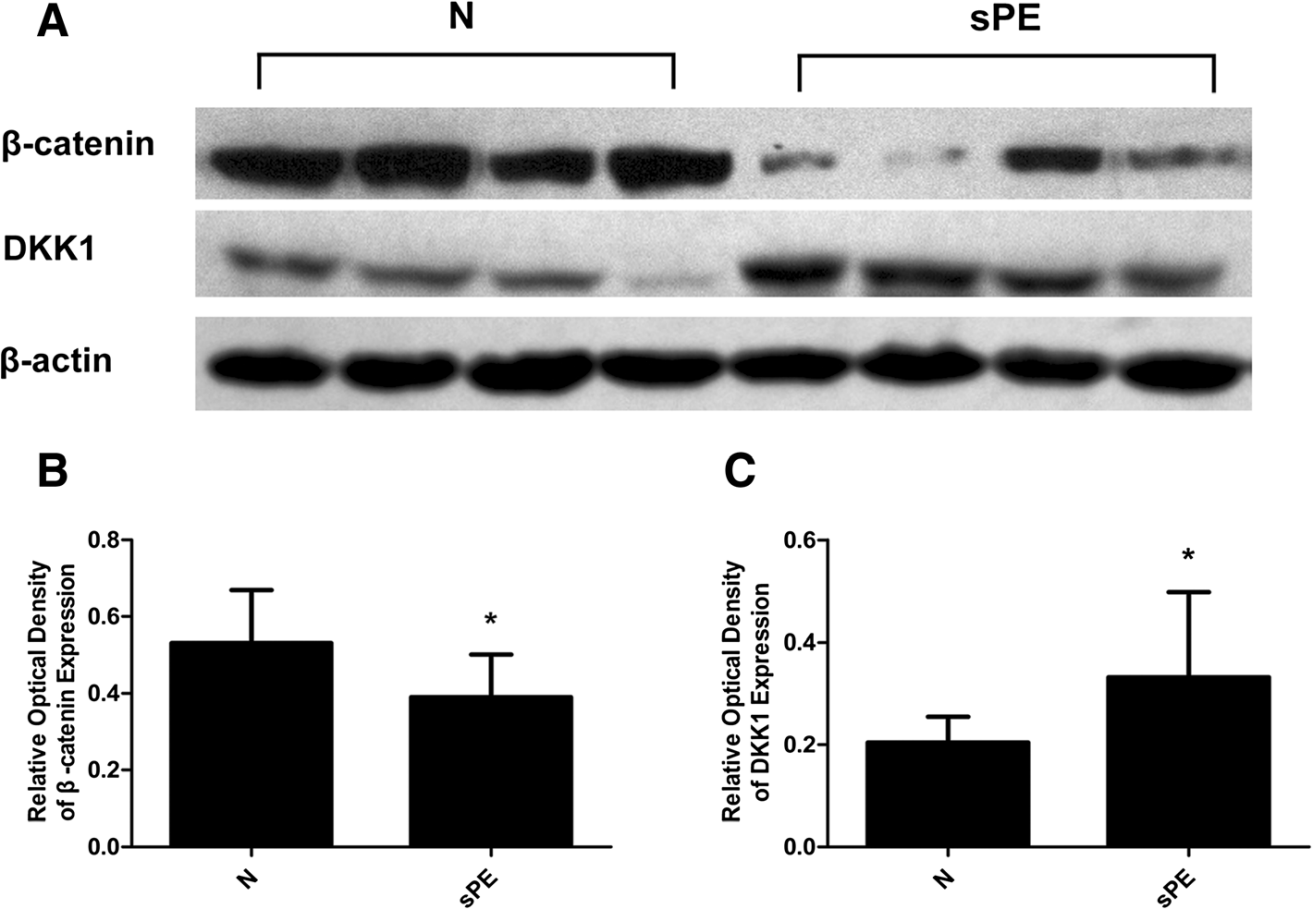
**The expression of β-catenin and DKK1 proteins in normal and severe preeclampsia group.** Figure 5**A**: Representative examples of β-catenin and DKK1 in placentas from normal control group (N) and severe preeclampsia group (sPE). The bar graph (**B**) and (**C**) shows the relative optical density of β-catenin and DKK1 expression, respectively. The β-catenin expression was significantly decreased, whereas the DKK1 was significantly increased in sPE group compared with the control group. (Data are presented mean ± SD, n=20 for each group, *p < 0.05, respectively).

We further confirmed our findings using western blot by evaluating the expression of β-catenin and DKK1 proteins from 20 normal and 20 preeclamptic placentas. The relative band densities for β-catenin and DKK1 expression after being normalized by β-actin expression are shown in Figure 5A. The results of immunostaining and western blot are consistent in that the β-catenin protein expression was significantly decreased in the severe PE group (0.39±0.11) compared with the normal control group (0.53±0.14)(P<0.05) (Figure 5B). However, the DKK1 expression was significantly increased in severe PE group (0.33±0.17) compared with the normal control group (0.20±0.05) (P<0.05) (Figure 5C).

### β-catenin and DKK1 expression in patients with preeclampsia who had an IUGR fetus and those with an AGA fetus

Moreover, we analysed theβ-catenin and DKK1 expression in patients with severe PE who had an IUGR fetus(the estimated fetal weight was below the 10th percentile) and those with an AGA fetus (the estimated fetal weight was between the 10th and 90th percentile). The β-catenin mRNA expression was decreased in the severe PE group who had an IUGR fetus (0.47±0.34) compared with those with an AGA fetus (0.76±0.40) (IUGR=11, AGA=19) (P<0.05). The β-catenin protein expression was decreased in the severe PE group who had an IUGR fetus (0.34±0.12) compared with those with an AGA fetus (0.43±0.09) (IUGR=9, AGA=11) (P<0.05). However, the DKK1 mRNA expression was increased in the severe PE group who had an IUGR fetus (2.35±0.86) compared with those with an AGA fetus (1.64±0.54)(P<0.05). The DKK1 protein expression was increased in the severe PE group who had an IUGR fetus (0.42±0.19) compared with those with an AGA fetus (0.27±0.13) (P<0.05).

Besides, we analysed the β-catenin and DKK1 expression between patients with preterm and term preeclampsia. However, the results suggested that there was no difference in the β-catenin mRNA expression between patients with preterm (0.56±0.31) and term PE(0.67±0.45) (preterm PE=10, term PE =20) (P>0.05). There is was no difference in the β-catenin protein expression between patients with preterm (0.36±0.12) and term PE(0.41±0.11) (preterm PE=8, term PE =12) (P>0.05). The DKK1 mRNA expression was no difference between patients with preterm (2.14±0.61) and term PE (1.97±0.94) (P>0.05). The DKK1 protein expression was no difference between the preterm (0.35±0.20) and term PE (0.31±0.13) (P>0.05).

## Discussion

The Wnt signaling is a complex pathway which is composed of the Wnt protein ligand, membrane receptors, extracellular inhibitory factors, intracellular signal switch and nuclear transcription factors. Wnt signaling has been identified as an essential pathway which is involved in development, tissue regeneration and cancer [16]. Sonderegger et al. analyzed the human Wnt ligands and their receptors in the human placenta, they found that 14 out of 19 Wnt ligands and 8 out of 10 receptors were detectable in human placental tissues [10]. Furthermore, Wnt3A stimulated trophoblast migration and invasion through the matrigel, suggesting that the canonical Wnt pathway may promote invasive of trophoblasts, and the exaggerated activation of the pathway could contribute to trophoblastic hyperplasia and local invasion [11]. In conclusion, recent studies have shown that the Wnt signaling pathway may regulate the processes of implantation and invasion. However, abnormal trophoblast invasion of spiral arterioles is believed to lead to PE. We speculated that undue inhibition of the Wnt signaling pathway in placentas might be involved in the pathogenesis of PE.

β-catenin is considered to be the intracellular signal switch in the canonical Wnt signaling pathway. It has been implicated in the genesis of many human cancers, including non-small cell lung cancer, colorectal carcinoma and many others cancer variants [17-20].

Previous study reported that the β-catenin destabilization could be provoked and trophoblast motility reduced because of the gene silencing of protease activated receptor-1, PAR1 [21]. Moreover, recent studies revealed that nuclear β-catenin expression in a considerable number of invasive trophoblasts in vivo, as well as after in vitro differentiation from chorionic villous explant cultures. Elevated numbers of β-catenin-positive nuclei were detected in the invasive trophoblasts of complete hydatidiform mole (CHM) placenta, suggesting that the abnormal activation of canonical Wnt signaling could play a pivotal role in the gestational disease [11]. Furthermore, early in pregnancy enhanced Wnt/β-catenin signaling is prerequisite for proper implantation and invasion of trophoblast cells [22]. The human placenta undergoes high levels of both angiogenesis and vasculogenesis in pregnancy, the initiation, maturation, and maintenance of the placental vasculature are crucial to the normal pregnancy. However, in the process of the pathogenesis of PE, the major pathological abnormality in the placenta is insufficient maternal spiral artery remodeling, cytotrophoblast cells fail to acquire the endothelial-like phenotype and are unable to invade the myometrial spiral arteries, this failure results in persistent placental hypoxia and dysfunction [23]. The recent study reported that vascular defects appeared in endothelial β-catenin −/− mutants mice, in the placenta of the endothelial β-catenin −/− mutants mice, the labyrinthine layer was reduced in thickness and less vascularized [24]. In this study, the results of immunostaining, real-time PCR and western blot are consistent in that the β-catenin expression was significantly decreased in the severe PE group. Thus, we speculated that the decreased expression of β-catenin may have certain associations with PE. The early placental development requires more maternal blood supply. This requirement is contented with the extensive remodeling of the maternal uterine spiral artery. Vascular remodeling depends on EVT, which has biological similarity to the behavior of tumor cells. However, in PE, the invasion of EVT and the placental circulation amount decreased. The expression of β-catenin in normal trophoblasts is reportedly localized at the membrane and cytoplasmatic compartment [25]. In this study, we observed that β-catenin localized at the syncytiotrophoblast and the EVT in both severe PE and control groups, however, the staining intensity of β-catenin in the placental tissue of the severe PE group was weaker than the normal controls group. However, the exact role of β-catenin requires further functional experiments.

DKK1, the founding member of the dickkopf (DKK) family, plays important roles in diverse developmental processes. It has been implicated in the process of tumorigenesis, such as in the case of colorectal cancer, medulloblastoma, and mesothelioma [26,27].

Recent studies have suggested that DKK1 may negatively affect the implantation and adhesion of the trophoblast to the endometrium. The treatment of JAR spheroids with DKK1 was shown to impair the attachment to endometrial-like Ishikawa cells [28]. DKK1 has also been reported to decrease the proliferation of cytotrophoblasts in human villous explants [11]. Furthermore, the treatment of primary trophoblasts and SGHPL-5 cells with DKK1 not only abolished Wnt-induced cell motility, but also reduced basal migration and invasion [29]. Our study indicated that DKK1 mRNA and protein expression was significantly increased in the severe PE group, furthermore, the staining intensity of DKK1 was greater in the severe PE group. Dickkopf-1 (DKK1) is a secreted glycoprotein that can antagonize the canonical Wnt signaling pathway, and our study indicated that the expression of DKK1 was increased in the placenta with severe PE, we speculated that the over-expression of DKK1 may have certain associations with PE, and the abnormal state of Wnt signaling pathway might be involved in the the pathogenesis of PE. Moreover, immunohistochemistry results showed that the DKK1 protein was primarily expressed in the syncytiotrophoblast, which is in accordance with previous studies. We also found that DKK1 was localized at the EVT. However, previous study reported that DKK1 was expressed in mice decidual cells [30], but in this study, we haven’t collect decidual specimen from human, the DKK1 expression in human decidual tissues and the exact role of DKK1 still need further study.

PE is a multisystem disease, the subclassifications of PE are often defined as: mild, moderate, and severe, as well as early and late. The concept of early and late PE is more modern, and it is widely accepted that these two entities have different etiologies and should be regarded as different forms of the disease [31,32]. In our hospital, we often choose conservative treatment, not cesarean delivery, for the pregnant woman with early- preeclampsia, so there is a limitation in obtaining the placenta tissue of these patients, however, in our future study, we expect that we could obtain variety of specimens including the mother blood and placenta of early and late PE, and do some research on the Wnt signaling in the early and late PE.

Clinical risk factors for intrauterine fetal growth contain severe PE, cardiopulmonary system disorders, umbilical cord and placenta dysfunction, etc. In this study, we also found that the β-catenin expression was decreased, and the DKK1expression was increased in the severe PE group who had an IUGR fetus compared with those with an AGA fetus (P<0.05). However, the results suggested that there is no difference in the expression of β-catenin and DKK1 between patients with preterm and term PE. Potential reasons for this finding may be that the premature birth contains spontaneous premature delivery and therapeutic premature delivery, the influence factors are complex.

## Conclusions

In summary, the pathogenesis of PE is highly complex, and the results of this study indicated that β-catenin and DKK1 are expressed in human third trimester placentas, decreased β-catenin expression, as well as DKK1 over-expression might be involved in the process of the pathogenesis of PE. Further studies would elucidate their exact roles in the pathogenesis of PE. Moreover, the expression of β-catenin and DKK1 during the first and second trimester, as well as the potential roles of the other associated proteins in Wnt signaling pathway in PE, awaits further study.

## Abbreviations

DKK1: Dickkopf-1; EVT: Extravillous trophoblast; H&E: Hematoxylin and eosin; IHC: Immunohistochemistry; LEF-1: Lymphoid enhancer-binding factor-1; PE: Preeclampsia; TCF: T cell-specific factors.

## Competing interests

The authors declare that they have no competing interests.

## Authors’ contributions

ZZ and HL participated in designing this study, writing and modifying this article, accomplishing the entire experiments. LLZ participated in collecting the samples, helping to accomplish the experiments. JLT and WP participated in helping to accomplish the experiments. All authors read and approved the final manuscript.
